# Identification of new HLA-A*0201-restricted cytotoxic T lymphocyte epitopes from neuritin

**DOI:** 10.1007/s11060-013-1167-6

**Published:** 2013-06-11

**Authors:** Zhao Yang, Tianzhi Zhao, Yong Liu, Zili Gong, Saiyu Cheng, Qingwu Yang

**Affiliations:** 1 Department of Neurology, Xinqiao Hospital, Third Military Medical University, Chongqing, 400037 China; 2 Department of Neurosurgery, Tangdu Hospital, Fourth Military Medical University, Xi’an, 710038 Shanxi China

**Keywords:** HLA-A*0201, Cytotoxic T lymphocyte, Epitopes, Neuritin

## Abstract

Identification of cytotoxic T lymphocyte (CTL) epitopes from additional tumor antigens is essential for the development of specific immunotherapy of malignant tumors. Neuritin, a recently discovered antigen overexpressed in astrocytoma, is considered to be a promising target for biological therapy. In the present study, we predicted and identified HLA-A2-restricted CTL epitopes from neuritin by using the following four-step procedure: (1) computer-based epitope prediction from the amino acid sequence of neuritin; (2) peptide-binding assay to determine the affinity of the predicted peptide with HLA-A2.1 molecule; (3) stimulation of primary T cell response against the predicted peptides in vitro; and (4) testing of the induced CTLs toward target cells expressing neuritin and HLA-A2.1. The results demonstrated that effectors induced by peptides of neuritin containing residues 13–21, 121–129 and 4–12 could specifically-secrete interferon-γ and lyse target cells. Our results indicate that these peptides are new HLA-A2.1-restricted CTL epitopes, and may serve as valuable tools for astrocytoma immunotherapy.

## Introduction

Astrocytoma is a common malignant primary brain tumor with a poor clinical prognosis in both adults and children [1–3]. The therapeutic options for the treatment of patients with astrocytoma are limited to three fundamental modalities: surgical resection, chemotherapy, and radiation therapy [4–6]. Cellular adoptive immunotherapy with specific CTLs has been recently used to treat malignant tumors [7–9].

Neuritin has been cloned and characterized as an important neurotrophin [10]. Recently, neuritin expression was found in astrocytomas and the expression level increased with astrocytoma pathologic grade, indicating that neuritin has an important role both in the promotion and in the progression of astrocytomas [11].

Anti-tumor vaccination is based on the existence of antigens, selectively or preferentially expressed by tumors, called tumor-associated antigens (TAAs) [12–14]. Vaccination with peptides derived from TAAs designed to stimulate specific T-cells is being a practicable approach evaluated in clinical trials [15–17]. To investigate the immune response elicited by neuritin CTL epitopes, the present study was undertaken to identify candidate CTL epitopes derived from neuritin.

## Materials and methods

### Cell lines and animals

The human TAP-deficient T2 cell line, BB7.2 cell line producing mAb against HLA-A2, human glioma cell line U251 and U87 (HLA-A2+), human breast cancer cell line MCF-7 (HLA-A2+) were purchased from the American Type Culture Collection (Manassas, VA). Cells were cultured in RPMI-1640 medium containing 10 % FBS (Gibco, with endotoxin level ≤ 10 EU/ml), penicillin (200 U/ml), and streptomycin (100 μg/ml). All cell lines mentioned previously were kept at 37 °C in a humidified atmosphere containing 5 % CO_2_. HLA-A*0201/Kb transgenic (Tg) mice, 8–12 weeks-old, were purchased from The Jackson Laboratory (USA). Mice were bred and maintained in specific pathogen-free (SPF) facilities. Animal experiments were performed in accordance with the guidelines of the Animal Care and Use Committee of Third Military Medical University.

### Peptide synthesis

In the present study, two programs BIMAS (http://www-bimas.cit.nih.gov/molbio/hla_bind/) and SYFPEITH (http://www.syfpeithi.de/Scripts/MHCServer.dll/EpitopePrediction.htm) were used to identify the candidate HLA-A2 restricted CTL epitopes from the neuritin antigen. The candidate peptides validated by epitope prediction were then synthesized by Fmoc chemistry (Sangon, China), and purified by HPLC to a purity of >95 %. Lyophilized peptides were dissolved in DMSO at a concentration of 20 mg/ml and stored at −70 °C. The control peptide HBcAg (18–27) (FLPSDFFPSV) was synthesized and purified using the same methodology.

### Peptide-binding assay

To determine whether the candidate epitopes can bind to HLA-A*0201 molecules, up-regulation of peptide-induced HLA-A*0201 molecules on T2 cells was examined. Briefly, according to previous reference, 1 × 10^6^ T2 cells were incubated with 50 μM of the synthesized peptides in serum-free RPMI 1640 medium supplemented with β_2_-microglobulin (Sigma) at a concentration of 3 μg/ml for 16 h at 37 °C, 5 % CO_2_ [18]. Expression of HLA-A*0201 on T2 cells was then determined with the FACS Calibur flow cytometer (Becton–Dickinson, USA), by staining with primary anti-HLA-A2 Ab derived from BB7.2 and FITC-labeled goat-antimouse IgG (BD Biosciences Pharmingen, USA) secondary antibody. The data were analyzed using Cell Quest software (Becton–Dickinson, USA). The Fluorescence index (FI) was calculated as follows: FI = (mean FITC fluorescence with the given peptide − mean FITC fluorescence without peptide)/(mean FITC fluorescence without peptide). Samples were measured in triplicate and then mean FI was calculated. An octapeptide mHpa (519–526) (FSYGFFVI) derived from mouse Hpa was served negative control.

### Measurement of the peptide/HLA-A*0201 complex stability

Briefly, T2 cells (10^6^/ml) were incubated overnight with the candidate peptides, respectively, at a concentration of 20 μg/ml in serum-free medium supplemented with β_2_-microglobulin at a concentration of 3 μg/ml at 37 °C.

Thereafter, they were washed four times to remove free peptides, incubated with Brefeldin A (10 lg/ml) for 1 h to block cell surface expression of newly synthesized HLA-A2.1 molecules, washed and incubated at 37 °C for 0, 2, 4, 6, or 8 h. Subsequently, cells were stained with anti-HLA-A2 antibody from BB7.2 cells to evaluate the HLA-A2.1 molecule expression. For each time point, peptide induced HLA-A*0201 expression was evaluated by the formula mentioned above. Dissociation complex50 (DC50) was defined as the time required for the loss of 50 % of the HLA-A*0201/peptide complexes stabilized at time = 0.

### RT-PCR analysis of neuritin expression

RT-PCR was used to analyze the expression of neuritin mRNA in cell lines. Total RNA was isolated from tumor cell lines using Tripure Isolation Regent Kit (Progema). Synthesis of cDNA was performed with 2 μg of total RNA with the aid of a reverse transcriptase Kit (Progema) and oligo(dT) primers. Two microliters RT product was amplified with PCR by using TaqDNA polymerase (Sangon, Shanghai) using standard procedures. The forward and reverse primer sequences were as follows: neuritin: sense primer, 5′-GTG CGA TGC AGT CTT TAA GTT-3′; anti-sense primer, 5′-GGG CTT TTC AGA CTG TTT GTT-3′; GAPDH: sense primer, 5′-GCA CCG TCA AGG CTG AGA AC-3′; antisense primer, 5′-ATG GTG GTG AAG ACG CCA GT-3′. Thirty amplification cycles were run: 1 min at 94 °C; 1 min at 60 °C; and 1 min at 72 °C. Cycling was ceased with a final extension of 10 min at 72 °C. RT-PCR products were then run on a gel and visualized with ethidium bromide.

### Western blot analysis of neuritin expression

For Western blot analysis, proteins in the cell extracts were separated by sodium dodecyl sulfate–polyacrylamide gel electrophoresis (SDS-PAGE) through an 8 % polyacrylamide gel and were then transferred onto a nitrocellulose membrane. The membrane was incubated with 5 % non-fat milk in PBS and later with anti-neuritin MAb for 2 h at room temperature. After washing, the membranes were incubated with an alkaline phosphatase-conjugated goat anti-mouse IgG antibody (Amersham Biosciences, Buckinghamshire, England) for 1 h at room temperature. Immunoreactive bands were detected using the ECL Western blot analysis system (Amersham Biosciences, Buckinghamshire, England).

### Dendritic cell generation from human peripheral blood precursors

PBMCs were isolated from healthy HLA-A2+ donors by Ficoll–Hypaque density gradient centrifugation (TBC company, Tianjing, China) and then seeded into culture flasks in RPMI-1640 medium supplemented with penicillin (100 U/ml), streptomycin (100 μg/ml), and 10 % FBS. After monocytes adhered (incubation for 2 h), the nonadherent cells were collected and frozen in freeze medium (60 % RPMI-1640 and 30 % FBS, 10 % DMSO) for later use in CTL assays. The adherent cells were cultured for 5 days in RPMI-1640 containing 1,000 U/ml of granulocyte–macrophage colony-stimulating factor (R&D Systems, Inc., Minneapolis, MN) and interleukin-4 (IL-4; R&D Systems, Inc.) and were the cultured for an additional 2 days in the presence of 1,000 U/ml of tumor necrosis factor α (R&D Systems, Inc.) to induce final maturation. After 7 days of culture, the mature DCs were harvested and analyzed for DC typical phenotypes by FACS analysis.

### Induction of peptide-specific CTL with synthetic peptides

Briefly, DCs were loaded with different peptides at a final concentration of 100 μg/ml for 4 h and were then irradiated with 20 Gy, which prevented all outgrowths in the control cultures. Autologous T cells were restimulated every 7 days with the previously mentioned peptide-pulsed DCs to generate peptide-specific CTLs. Recombinant interleukin 2 (IL-2) at a concentration of 20 U/ml was added to the culture medium on day 3 after every stimulation. Cytotoxic T lymphocyte activity was then assessed on day 23 by a 4 h ^51^Cr release assay. Effectors generated from negative peptide-pulsed DCs were used as controls.

### ELISPOT assay

IFN-γ secretion of effectors was assayed by enzyme-linked immunospot (ELISPOT). Multiscreen 96-well assay plates (Dakewe, Shenzhen, China) were precoated overnight at 4 °C with anti-IFN-γ antibody according to the manufacturer’s instruction. After washing with PBST (PBS-0.05 % Tween 20), plates were blocked for 1 h at 37 °C with PBS/1 % BSA. Cytotoxic T lymphocyte effectors from human HLA-A2+ donors were plated in triplicate wells at a density of 1 × 10^5^/100 μl in RPMI-1640 medium. Plates were cultured overnight, washed extensively with PBST, and incubated with anti-IFN-γ mAb for 1 h at 37 °C. After washing, goat anti-biotin antibodies (Dakewe) were added, and the plates were incubated for 1 h at 37 °C. Thirty microliters of activator solution (Dakewe) was added to develop spots, and after 10–30 min, the plates were washed with distilled water to stop the reaction. After being air-dried, the number of spots in each well was counted using the Bioreader 4000 PRO-X (Bio-Sys; Germany).

### Cytotoxicity assay

To evaluate the levels of CTL activity, a standard 4-h ^51^Cr release assay was used. Briefly, target cells were incubated with ^51^Cr (100 μCi per 1 × 10^6^ cells) for 2 h in a 37 °C water bath. After incubation with ^51^Cr, target cells were washed three times with PBS, resuspended in RPMI-1640 medium, and mixed with effector cells at a 25:1, 50:1 or 100:1 of effector to target (E/T) ratio. Assays were performed in triplicate for each sample at each ratio in a 96-well round-bottomed plate. After a 4-h incubation, the supernatants were harvested, and the amount of released ^51^Cr was measured with a gamma counter. The percent specific lysis was calculated according to the following formula:

Specific lysis = (experimental release − spontaneous release)/(maximal release − spontaneous release) × 100 %

### Analysis of in vivo immunogenicity

HLA-A*0201/Kb mice were immunized with 100 μg of various peptides prepared in incomplete Freund’s adjuvant (IFA) and boosted once a week for three times. As a control, mice were injected with an IFA emulsion without peptide. 7 days after immunization, splenocytes from injected animals were cultured and used as effector cells.

### Statistical analysis

Results were expressed as mean ± SEM. Analysis of Student’s *t* test were performed to determine effects of the treatments. A difference was considered significance level of *P* < 0.05.

## Results

### Prediction of putative CTL epitopes restricted with HLA-A*0201

To predict the HLA-A*0201-restricted CTL epitopes of neuritin, two programs (BIMAS and SYFPEITHI), were used to scan the complete amino acid sequence of this antigen. Four highest-scored 9-amino-acid peptides were chosen as candidates for further identification (Table 1). These peptides were chemically synthesized, purified, and identified. The molecular weight of each peptide determined by mass spectrometry assay was similar to its theoretical molecular weight, and the purities of these peptides were all >95 % (data not shown).

**Table 1:** Predicted neuritin epitopes binding to HLA-A2.1

Position	Length	Sequence	BIMAS score	SYFPEITHI score
13–21	9	ILAVQIAYL	459.398	30
121–129	9	LLPAFPVLL	138.001	25
4–12	9	KLNGRYISL	112.153	26
127–135	9	VLLVSLSAA	71.872	25

### MHC peptide-binding and stability assay

The binding affinity and stability of these peptides to HLA-A2.1 was determined by using antigen processing-deficient T2 cells because their enhanced HLA-A2.1. As shown in Table 2, all of the peptides synthesized were bound to HLA-A2.1 molecules but with different affinity and stability. Of four peptides selected, neuritin_13–21_ up-regulated the HLA-A2.1 molecular expression and showed high affinity and stability to HLA-A2.1, whereas neuritin_121–129_ and neuritin_4–12_ showed moderate affinity and neuritin_127–135_ only had low affinity and stability to the molecule.

**Table 2:** HLA-A2-binding affinity and stability of neuritin-derived peptides

Position	Length	Sequence	FI	DC50
13–21	9	ILAVQIAYL	1.68	>8
121–129	9	LLPAFPVLL	1.24	6–8
4–12	9	KLNGRYISL	1.07	6–8
127–135	9	VLLVSLSAA	0.68	2–4
519–526	9	FSYGFFV	0.38	<2

The shaded region is the control peptide values

### Expression of neuritin in target cells

**Fig. 1:**
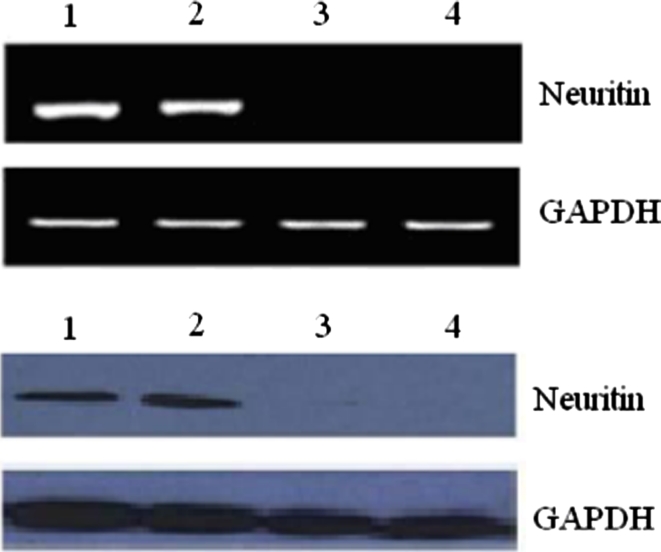
Expression of neuritin in different target cells. Total RNA was isolated from tumor cell lines using Tripure Isolation Regent Kit. Two microliters RT product was amplified with PCR by using TaqDNA polymerase (using standard procedures). RT-PCR products were then run on a gel and visualized with ethidium bromide. For Western blot analysis, proteins in the cell extracts were separated by SDS-PAGE and were then analyzed with anti-neuritin MAb (antibodies-online company). *1* U251 cells; *2* U87 cells; *3* MCF-7 cells; *4* autologous lymphocytes

The expression of neuritin mRNA and protein in cell lines in this study was analyzed by RT-PCR and Western blot. As shown in Fig. 1, neuritin mRNA and protein were detected in U251 and U87 cell lines. However, neuritin mRNA and protein could not be detected in MCF-7 and autologous lymphocytes.

### Enzyme-linked immunospot (ELISPOT) assay for IFN-γ

**Fig. 2:**
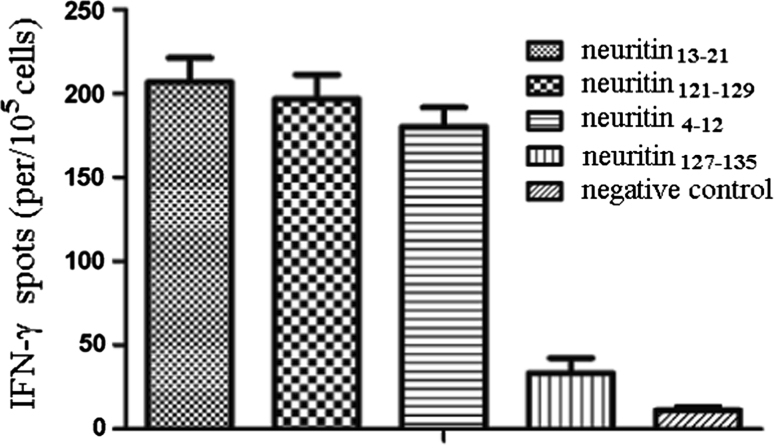
Specific IFN-γ by ELISPOT assay. The PBMCs of human HLA-A2+ donors were obtained and then cultured in RPMI 1640 supplemented with 10 % FCS, 100 U/ml penicillin, and 100 μg/ml streptomycin. Dendritic cell were generated, and loaded with different peptides at a final concentration of 100 μg/ml for 4 h and were then irradiated with 20 Gy, which prevented all outgrowths in the control cultures. Autologous T cells were restimulated every 7 days with the peptide-pulsed DCs to generate peptide-specific CTLs. The IFN-γ secretion was then assessed on day 23. Experiments performed in triplicate showed consistent results. Compared with controls, *P* < 0.05

Since CTLs are known to produce the Th1 cytokine IFN-γ, peptide-specific T cells were enumerated by measuring IFN-γ-producing cells by ELISPOT assay. As shown in Fig. 2, neuritin_13–21_, neuritin_121–129_ and neuritin_4–12_ peptides were found to generate a strong peptide-specific T cell response by virtue of their ability to induce increased frequencies of IFN-γ-producing T cells, as compared to the negative peptide control. These results suggest that neuritin peptide vaccines can increase IFN-γ secretion by effectors and enhance the Th1 immune response.

### Induction of CTLs efficiently in vitro

**Fig. 3:**
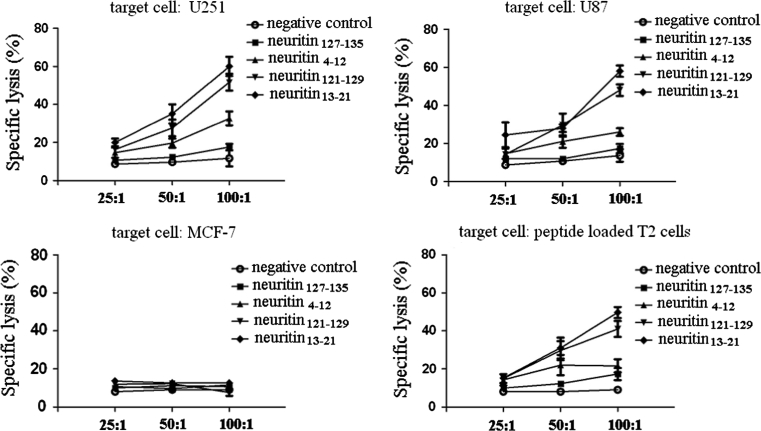
Specific lysis of CTLs against target cells. Target cells were incubated with ^51^Cr (100 μCi per 1 × 10^6^ cells) for 2 h in a 37 °C water bath. After incubation with ^51^Cr, target cells were washed three times with PBS, resuspended in RPMI-1640 medium, and mixed with effector cells at a 25:1, 50:1 or 100:1 of effector to target (E/T) ratio. After a 4-h incubation, the supernatants were harvested, and the amount of released ^51^Cr was measured with a gamma counter. Compared with controls, *P* < 0.05

PBMCs from four HLA-A2.1+ donors were stimulated with synthetic peptides with the previously published method for CTL induction [19]. Of four tested, neuritin_13–21_, neuritin_121–129_ and neuritin_4–12_ peptides were able to elicit neuritin-specific CTLs, which could lyse target cells expressing neuritin and HLA-A2.1 (Fig. 3).

### Inhibition of the recognition of effectors by anti-HLA2 antibody

**Fig. 4:**
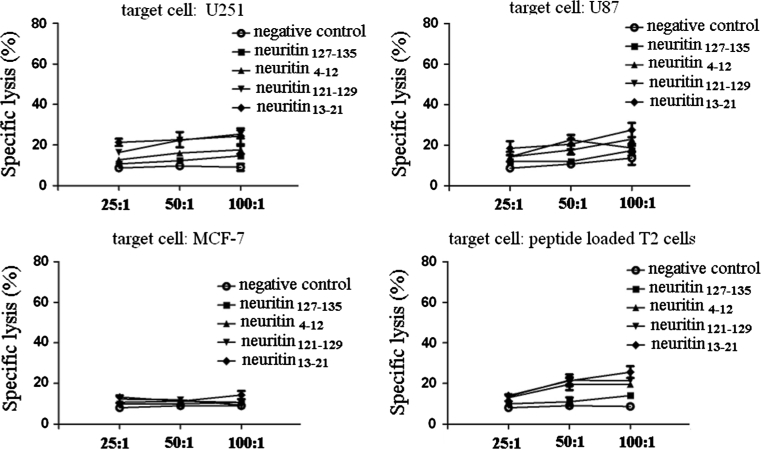
Inhibited recognition of induced cells by anti-HLA-A2 antibody. Target cells were incubated with 100 μl anti-HLA-A2 antibody (functionally blocking mAb, from BB7.2 cell hybridoma supernate) for 1 h at 4 °C. The cytotoxic activities of CTLs were determined against target cells at various E/T ratios using ^51^Cr release assay. Experiments performed in triplicate showed consistent results. Compared with controls, *P* < 0.05

To determine whether the peptides induced effectors recognized target cells in an HLA-A2-restricted manner, the mAbs against HLA-A2 were used to block recognition by effectors. Our results showed that the anti-HLA-A2 antibody could significantly eliminate the cytotoxicity of the effectors against neuritin and HLA-A2 positive cells (Fig. 4), which implied that the induced effectors lysed target cells in an HLA-A2-restricted manner.

### In vivo induction of epitope-specific CTLs in vivo

**Fig. 5:**
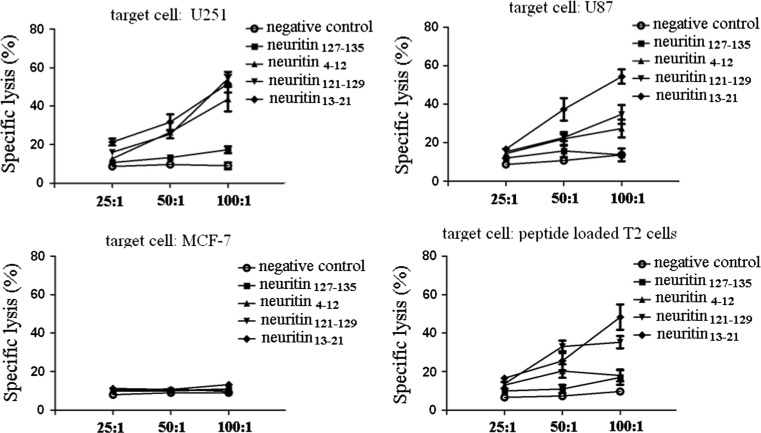
In vivo induction of epitope-specific CTLs in vivo. HLA-A*0201/Kb mice were immunized with 100 μg of various peptides prepared in incomplete Freund’s adjuvant (IFA) and boosted once a week for three times. As a control, mice were injected with an IFA emulsion without peptide. 7 days after immunization, splenocytes from injected animals were cultured and used as effector cells. The cytotoxic activities of CTLs were determined against target cells at various E/T ratios using ^51^Cr release assay. Experiments performed in triplicate showed consistent results. Compared with controls, *P* < 0.05

We investigated whether peptides could induce immunity in vivo. HLA-A*0201/Kb mice were immunized with 100 μg of various peptides prepared in incomplete Freund’s adjuvant (IFA). The cytolytic assay showed that CTLs primed from neuritin_13–21_, neuritin_121–129_ and neuritin_4–12_ immunized mice could lyse neuritin and HLA-A2.1 positive cells with high efficiency (Fig. 5). These results suggested that the peptides could also achieve higher immunogenicity in vivo.

## Discussion

Survival in the majority of high-grade astrocytoma (HGA) patients is very poor, with only a rare population of long-term survivors [20–22]. During the last decades, great progress has been made in the treatment of patients with astrocytoma [23–25]. Many astrocytoma can be cured or eliminated using the therapeutic options such as surgical resection, chemotherapy, and (or) radiation therapy. However, as for advanced astrocytoma, the modalities mentioned above do not yield good results [26–28].

Over the past few years, the analysis of spontaneous immune responses to autologous tumors in cancer patients has allowed the identification of several kinds of tumor-associated antigens that can be the targets for tumor specific immune responses based on the recognition of tumor antigen by CTLs in an MHC-class I/peptide complex-restricted manner [29–31]. Therefore, cancer-specific immunotherapy has become an attractive fourth-therapeutic approach against carcinomas. Among them, one of the most relevant for the development of tumor immunotherapy is peptide-based, cancer-specific immunotherapy using the group of the tumor antigens [32, 33].

Neuritin has been cloned and characterized as an important neurotrophin. The expression of neuritin is closely associated with the growth of afferent nerves and the development of dendrites, axons, and synapses [34]. Recently, neuritin expression was found in tissues besides nervous system and tumor tissues [35]. Moreover, the neuritin protein was highly expressed in astrocytomas and increased with pathologic grade, indicating that neuritin has an important role both in the promotion and in the progression of astrocytomas.

In this study, we first predicted four candidate epitopes from neuritin antigen by using HLA-A2.1-restricted epitope prediction algorithms based on long distance prediction systems SYFPEITHI and BIMAS. Secondly, peptide-binding assay was used to determine the affinity of every epitope with HLA-A2.1 and the results showed that neuritin_13–21_ had high affinity to HLA-A2.1, whereas neuritin_121–129_ and neuritin_4–12_ showed moderate affinity to the molecule. Thirdly, cytotoxic activity of CTLs was measured by ELISPOT and ^51^Cr release assay. The results demonstrated that neuritin_13–21_, neuritin_121–129_ and neuritin_4–12_ could elicit CTLs to lyse target cells in an HLA-A2.1-restricted manner. Lastly, we immunized the HLA-A*0201/Kb mice with various peptides and found neuritin_13–21_, neuritin_121–129_ and neuritin_4–12_ could also elicit CTLs to lyse neuritin and HLA-A2.1 positive target cells. These results suggested that the peptides had the potential of immunogenicity in vivo.

In conclusion, our results suggest that neuritin_13–21_, neuritin_121–129_ and neuritin_4–12_ might be capable of inducing HLA-A2.1-restricted CD8^+^ CTL, which would be lethal for neuritin and HLA-A2.1 positive cells. Therefore, identification of neuritin peptide would contribute to the design of epitope-based vaccine for astrocytomas immunotherapy.
